# Curcumin suppresses cell growth and invasion and induces apoptosis by down-regulation of Skp2 pathway in glioma cells

**DOI:** 10.18632/oncotarget.4090

**Published:** 2015-05-23

**Authors:** Lixia Wang, Xiantao Ye, Xingming Cai, Jingna Su, Renqiang Ma, Xuyuan Yin, Xiuxia Zhou, Huabin Li, Zhiwei Wang

**Affiliations:** ^1^ The Cyrus Tang Hematology Center and Collaborative Innovation Center of Hematology, Jiangsu Institute of Hematology, The First Affiliated Hospital, Soochow University, Suzhou, China; ^2^ Department of Geriatrics, The First Affiliated Hospital of Sun Yat-sen University, Guangzhou, China; ^3^ Department of ENT, Head and Neck Surgery, The First Affiliated Hospital of Sun Yat-sen University, Guangzhou, China

**Keywords:** curcumin, Skp2, glioma, growth, invasion

## Abstract

Studies have demonstrated that curcumin exerts its tumor suppressor function in a variety of human cancers including glioma. However, the exact underlying molecular mechanisms remain obscure. Emerging evidence has revealed that Skp2 (S-phase kinase associated protein 2) plays an oncogenic role in tumorigenesis. Therefore, we aim to determine whether curcumin suppresses the Skp2 expression, leading to the inhibition of cell growth, invasion, induction of apoptosis, and cell cycle arrest. To this end, we conducted multiple methods such as MTT assay, Flow cytometry, Wound healing assay, invasion assay, RT-PCR, Western blotting, and transfection to explore the functions and molecular insights of curcumin in glioma cells. We found that curcumin significantly inhibited cell growth, suppressed cell migration and invasion, induced apoptosis and cell cycle arrest in glioma cells. Furthermore, we observed that overexpression of Skp2 promoted cell growth, migration, and invasion, whereas depletion of Skp2 suppressed cell growth, migration, and invasion and triggered apoptosis in glioma cells. Mechanistically, we defined that curcumin markedly down-regulated Skp2 expression and subsequently up-regulated p57 expression. Moreover, our results demonstrated that curcumin exerts its antitumor activity through inhibition of Skp2 pathway. Collectively, our findings suggest that targeting Skp2 by curcumin could be a promising therapeutic approach for glioma prevention and therapy.

## INTRODUCTION

Glioma is one of the common primary brain tumors in adults. Due to its aggressive growth and invasion, the median survival is generally less than one and half years from the time of diagnosis [1]. Even in the favorable situations, most patients with glioma die within two years [1]. This high lethality could be due to that surgery cannot remove entire tumor without harming health brain. Standard treatment with temozolomide and radiotherapy has increased the median overall survival by 15–20 months [2]. However, chemotherapy treatment needs to overcome the blood-brain barrier and drug resistance. Despite progress in glioma therapy regimens such as surgery, radiation, chemotherapy, or combined modalities, the prognosis for malignant glioma patients remains dismal [3]. Thus, it is necessary to discover new potential therapeutic agents for the clinical studies.

A growing body of evidence implicates that curcumin, a golden pigment extracted from turmeric, exhibits pleiotropic activities such as antioxidant, anti-inflammatory, antiviral, antifungal, antibacterial, anti-diabetic, and neuroprotective properties [4]. Moreover, many studies have demonstrated that curcumin exerts its anti-tumor activity in a broad spectrum of human cancers including glioma [5–8]. For example, it has been reported that curcumin inhibited cell proliferation via targeting the Wnt/β-catenin signaling pathway in medulloblastoma [9]. Moreover, Du et al. found that curcumin inhibited SHH (sonic hedgehog)/Gli1 signaling pathway, leading to inhibition of cell growth and induction of apoptosis *in vitro* and *in vivo* in glioma [10]. Further study showed that curcumin exerted its antitumor activity involved in reactivation of RANK (receptor activator of nuclear factor κB) and inactivation of STAT3 (signal transducer and activator of transcription 3) in glioblastoma cells [11]. Notably, curcumin synergistically enhanced paclitaxel-mediated cell growth inhibition in glioma cells [12]. Additionally, curcumin was discovered to suppress the cell growth through inhibition of HADC4 (histone deacetylase 4) and NF-κB (nuclear factor kappa-B) pathways in medulloblastoma cells [13, 14]. Although many studies have revealed the molecular basis of curcumin-induced cell growth inhibition, the underlying molecular mechanisms have not been fully elucidated.

Skp2 (S-phase kinase associated protein 2) as a key oncoprotein has been characterized to play an oncogenic role in tumorigenesis [15–19]. Skp2 belongs to the ubiquitin proteosome system and exerts its oncogenic functions via degradation of its ubiquitination targets such as p21 [20], p27 [21], p57 [22], E-cadherin [23], and FOXO1 (forkhead box O1) [24]. Overexpression of Skp2 has been identified and is associated with poor prognosis in various types of human cancers [25, 26]. Lin et al. reported that Akt interacts with and directly phosphorylates Skp2, leading to promotion of cell proliferation and tumorigenesis [27]. This group also found that targeting Skp2 suppressed tumorigenesis through Arf-p53-independent cellular senescence [28]. Our previous study has shown that Skp2 is acetylated by p300 and subsequently promoted its cytoplasmic retention, which enhanced cell migration through degradation of E-cadherin [23, 29]. Chan et al. reported that the Skp2-SCF (Skp, cullin, F-box containing complex) E3 ligase activated Akt ubiquitination, herceptin sensitivity and tumorigenesis [30]. This group further identified that inhibition of Skp2-SCF ubiquitin ligase restricts cancer stem cell traits and cancer progression [31]. These studies indicate that inactivation of Skp2 could be a promising approach for treating human cancers [32].

In the current study, we determined whether overexpression of Skp2 promoted cell growth, migration and invasion, but induced cell apoptosis and cell cycle arrest. Moreover, we explored whether curcumin exhibits its anticancer activity via inactivation of Skp2 in glioma cells. Our results demonstrated that Skp2 was critically involved in glioma tumorigenesis and that curcumin down-regulated the expression of Skp2, resulting in upregulation of p57 and down-regulation of pAkt, which could lead to inhibition of tumorigenesis. Our findings suggest that curcumin could be a potential efficient agent for the treatment of glioma.

## RESULTS

### Curcumin inhibited cell proliferation

**Figure 1:**
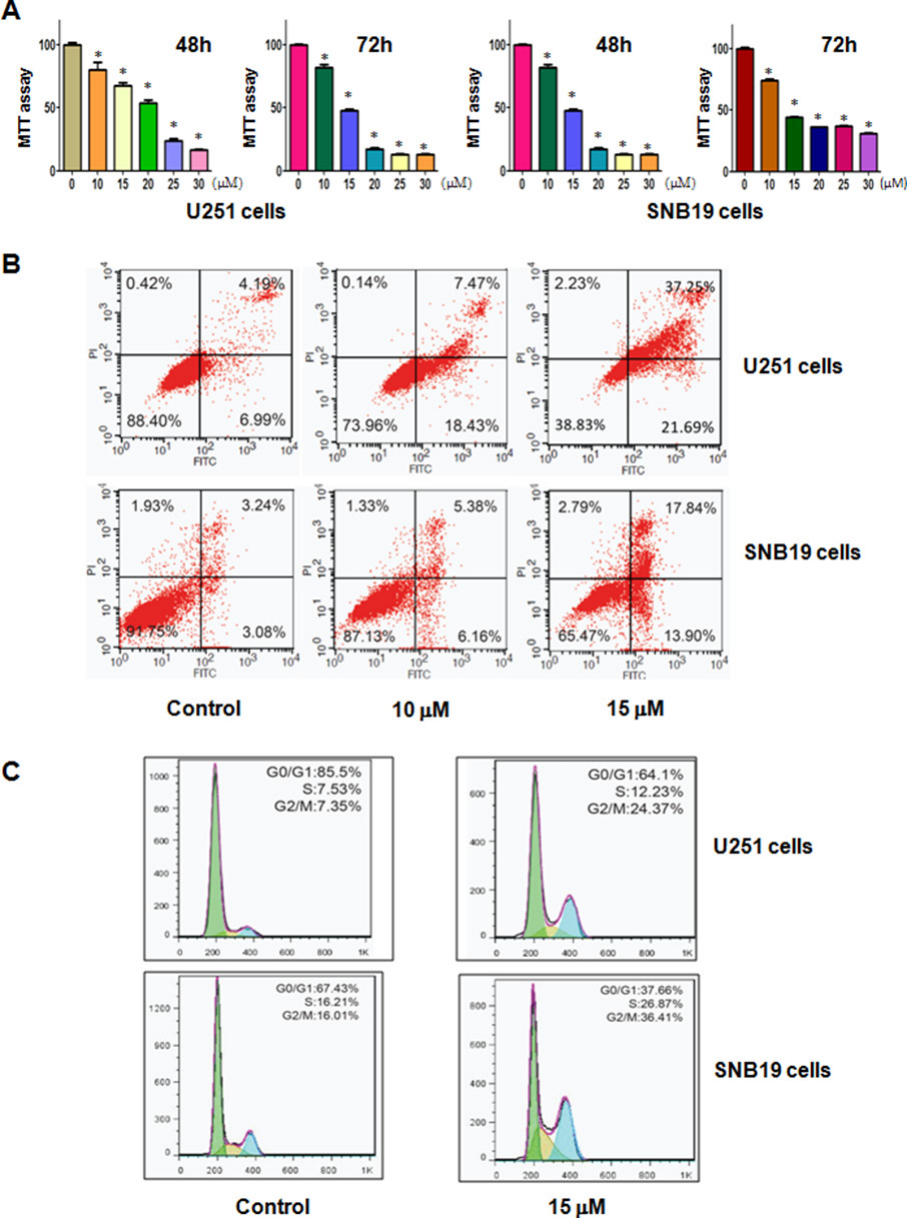
Effect of curcumin on cell growth, apoptosis, and cell arrest **A.** Effect of curcumin on cell growth in glioma cells was detected by MTT assay. *P < 0.05, compared to the control. **B.** Cell apoptosis in glioma cells treated with curcumin was determined by Flow cytometry. **C.** Curcumin induced glioma cell cycle arrest.

To detect whether curcumin treatment inhibits cell growth in glioma cells, MTT assay was used to measure the growth viability in U251 and SNB19 cells treated with different concentrations of curcumin for 48 hours and 72 hours, respectively. As expected, we found that curcumin significantly inhibited cell growth in time- and dose-dependent manner in both U251 and SNB19 cells (Figure 1A). The IC_50_ that caused 50% inhibition of cell growth at 72 hours for both glioma cell lines was found to around 15 μM (Figure 1A). Therefore, we used 15 μM curcumin in the following studies.

### Curcumin induced apoptosis

It has been known that curcumin-mediated cell growth inhibition could be due to the increased apoptosis. Thus, we further explored whether curcumin could trigger apoptosis in glioma cells. To achieve this goal, we detected the effects of curcumin treatment on apoptotic cell death using PI-FITC-annexin assay. U251 and SNB19 cells were treated with 10, 15 μM curcumin for 48 hours. After treatment, we measured the cell apoptosis and observed that the induction of cell apoptosis by curcumin treatment was dose-dependent (Figure 1B), suggesting that curcumin treatment led to apoptosis in glioma cells.

### Curcumin induced cell cycle arrest

To further define the anti-tumor effect of curcumin on glioma cells, we conducted the cell cycle analysis by PI staining and flow cytometry in U251 and SNB19 cells treated with 15 μM curcumin for 48 hours. We identified a typical G2/M arrest pattern from 7% with control to 24% with curcumin treatment in U251 cells (Figure 1C). Similarly, curcumin treatment caused G2/M arrest in SNB19 cells (Figure 1C). These results showed that curcumin distinctly caused G2/M phase arrest in glioma cells.

### Curcumin inhibited cell migration and invasion

**Figure 2:**
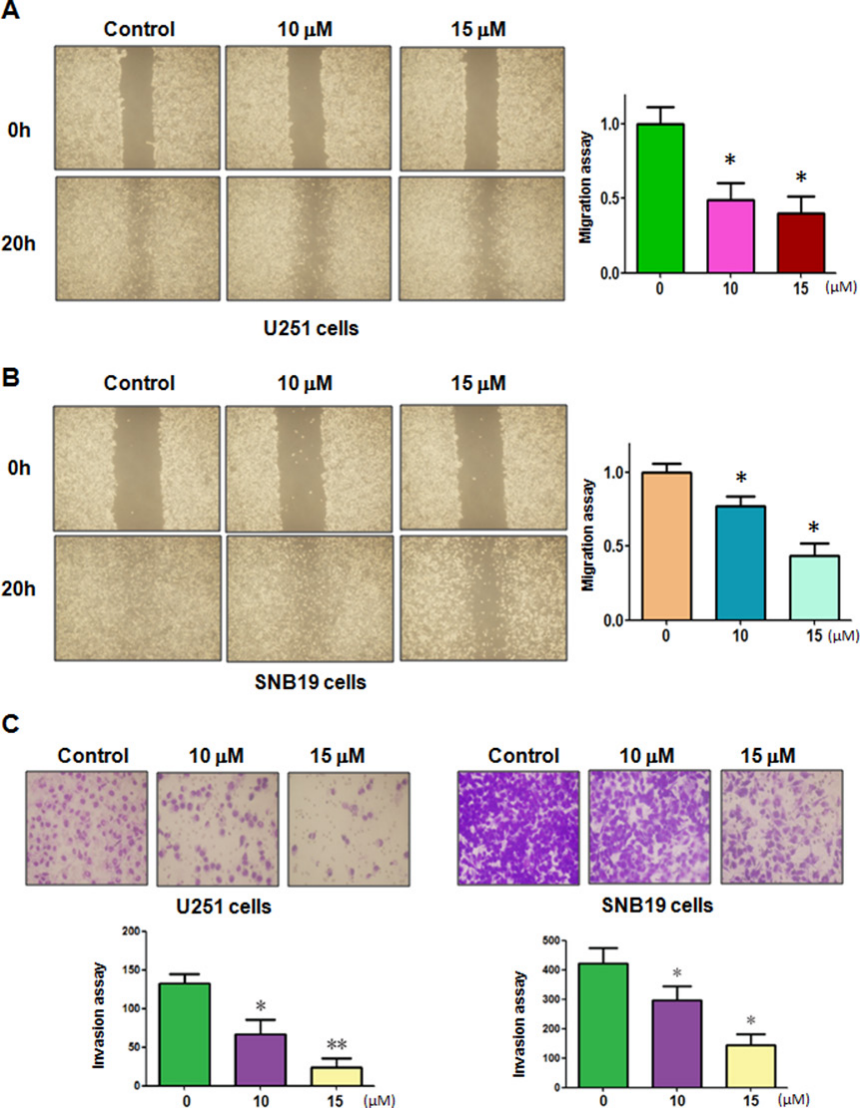
Curcumin inhibited cell migration and invasion on glioma cells **A–B.** The inhibitory effect of curcumin on glioma cell migration was detected using wound healing assay in U251 cells (A, left panel) and SNB19 cells (B, left panel). Right panel, Quantitative results are illustrated for left panels. *P < 0.05, vs control. **C.** Top panel, the inhibitory effect of curcumin on glioma cell invasion was detected by Transwell chambers assay. Bottom panel, Quantitative results are illustrated for Top panel. *P < 0.05, **P < 0.01 vs control.

To dissect whether curcumin inhibited the motility of the cells, we conducted the wound healing assay using scratch approach and invasion assay using matrigel-coated membrane. Our wound healing assay demonstrated that curcumin significantly decreased cell migration in both U251 and SNB19 cells (Figure 2A and 2B). Consistent with this result, curcumin treatment led to decreased penetration of glioma cells via the matrigel-coated membrane compared with the control cells (Figure 2C). Altogether, curcumin has anti-invasive function in glioma cells.

### Curcumin decreased Skp2 expression

**Figure 3:**
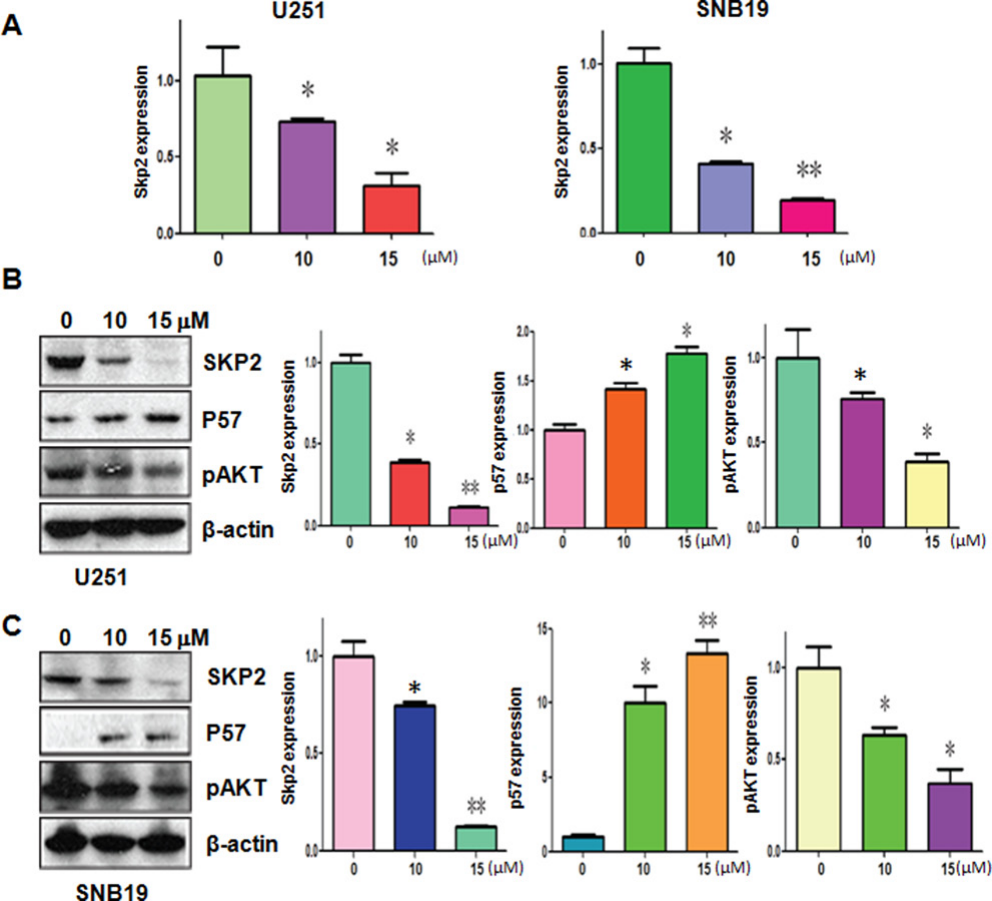
Curcumin downregulated Skp2 expression at RNA and protein levels **A.** The Skp2 mRNA expression was detected using RT-PCR in glioma cells treated with curcumin. *P < 0.05, **P < 0.01 vs control. **B–C.** Left panel, the expression of Skp2, pAkt and p57 was determined by western blotting analysis in U251 (B) and SNB19 cells (C) after curcumin treatment. Right panel, Quantitative results are illustrated for left panel. *P < 0.05, **P < 0.01, compared to the control.

Emerging evidence has revealed that Skp2 exerts its oncogenic functions in tumorigenesis. To determine the molecular mechanism of curcumin-mediated anti-tumor activities, we measured the expression of Skp2 at mRNA and protein levels in glioma cells treated with curcumin. Our real-time RT-PCR analysis results demonstrated that Skp2 mRNA levels were markedly decreased in U251 and SNB19 cells after curcumin treatment (Figure 3A). More importantly, our Western blotting analysis showed that curcumin also down-regulated the Skp2 protein level in both glioma cells (Figure 3B and 3C). To further validate the inactivation of Skp2 by curcumin could impact on Skp2 downstream target genes such as pAkt and p57, we detected the expression of pAkt and p57 in glioma cells with curcumin treatment. We found that curcumin down-regulated the expression of pAkt and p57 in both U251 and SNB19 cells (Figure 3B and 3C). Taken together, curcumin exerts its anti-tumor activity through down-regulation of Skp2 signaling pathway.

### Over-expression of Skp2 decreased curcumin-induced cell growth inhibition

**Figure 4:**
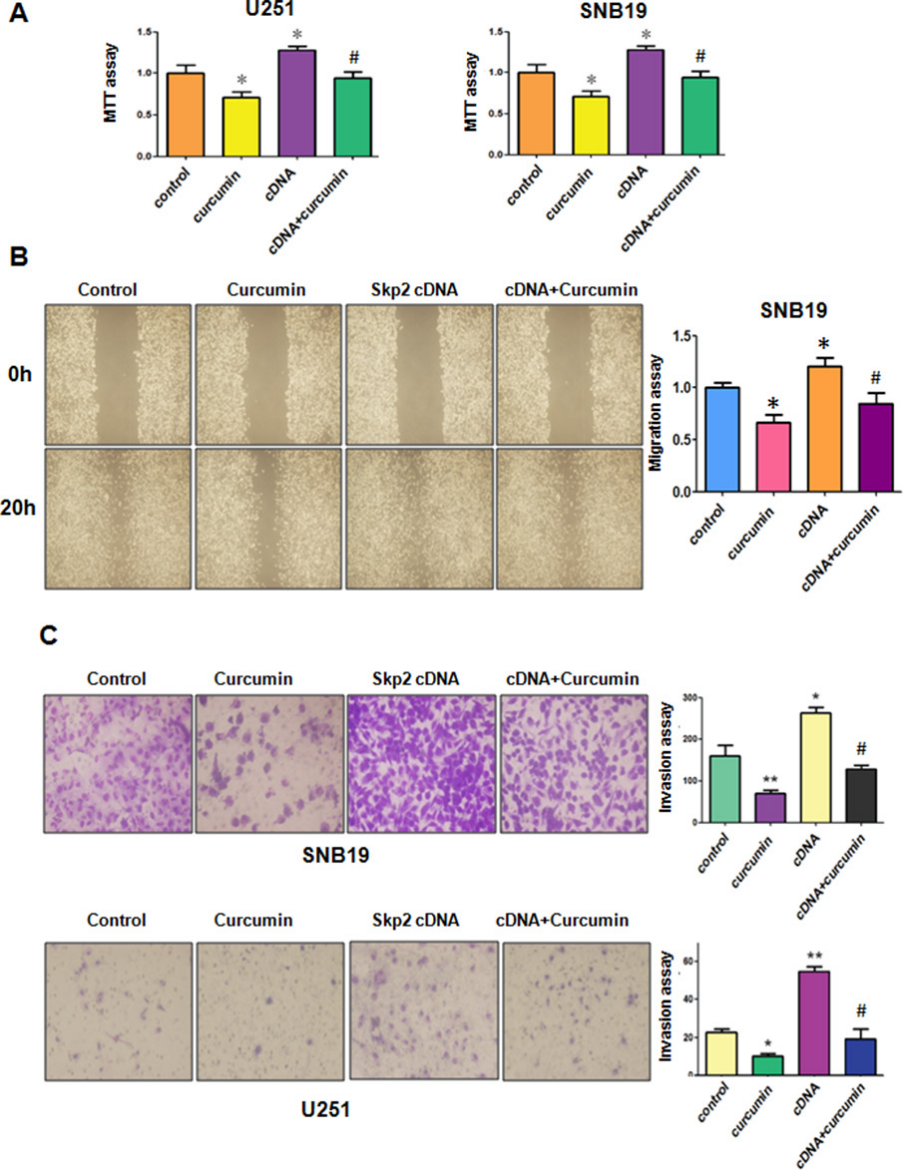
The effect of Skp2 overexpression on cell growth, migration and invasion **A.** MTT assay was used to detect the effect of Skp2 overexpression in combination with curcumin treatment on glioma cell proliferation. *P < 0.05, **P < 0.01, compared with control; ^#^*P* < 0.05 compared with curcumin treatment or Skp2 cDNA transfection. **B.** Left panel, The wound healing assay was conducted to detect the cell migration in glioma cells after Skp2 cDNA transfection and curcumin treatment. cDNA: Skp2 cDNA; cDNA+Curcumin: Skp2 cDNA+Curcumin. Right panel, Quantitative results are illustrated for left panel. **C.** Left panel, Invasion assay was performed in glioma cells after Skp2 cDNA transfection and curcumin treatment. Right panel, Quantitative results are illustrated for left panel.

To determine whether curcumin exhibits its anti-tumor activity partly through down-regulation of Skp2 in glioma cells, U251 cells and SNB19 cells were transfected with Skp2 cDNA or empty vector as control. We observed that over-expression of Skp2 promoted cell growth in both glioma cell lines (Figure 4A). Moreover, over-expression of Skp2 rescued cell growth inhibition by curcumin treatment in U251 cells and SNB19 cells (Figure 4A), suggesting that curcumin-mediated cell growth inhibition could be in part due to down-regulation of Skp2. These results suggest that curcumin exerts its biological function via inactivation of Skp2 in glioma cells.

### Over-expression of Skp2 enhanced glioma cell motility

**Figure 5:**
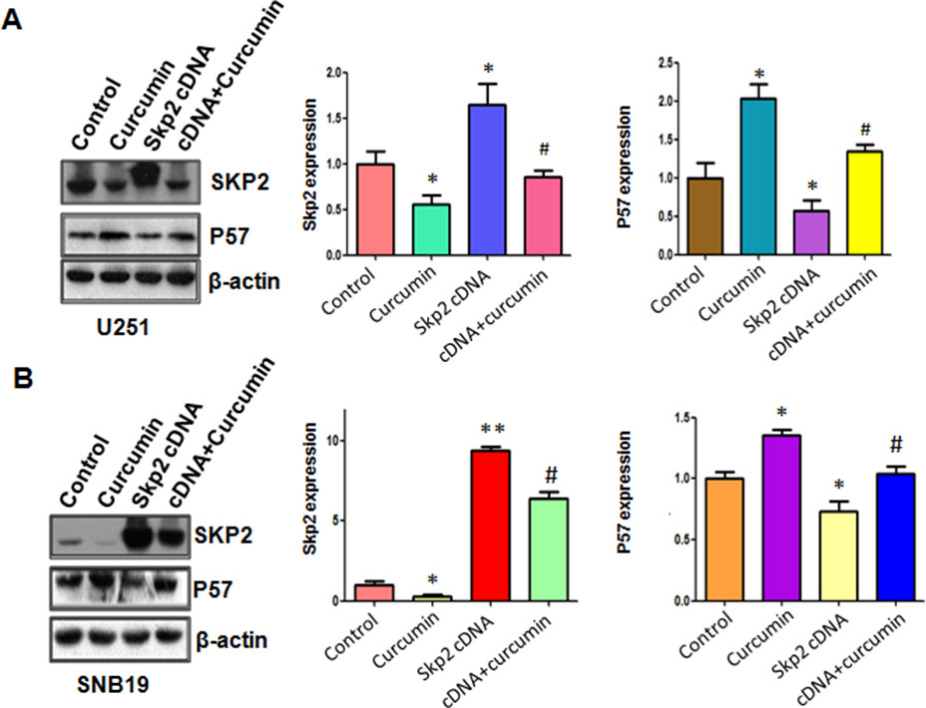
The expression of Skp2 and p57 was measured in Skp2 cDNA transfected glioma cells treated with curcumin **A–B.** Left panel: The expression of Skp2 and its target p57 was detected by western blotting in glioma cells with Skp2 cDNA transfection and curcumin treatment. Right panel: Quantitative results are illustrated for left panel. *P < 0.05, **P < 0.01, compared with control; ^#^*P* < 0.05 compared with curcumin treatment or Skp2 cDNA transfection.

Next, we explored whether Skp2 could control cell motility in glioma cell lines. The results from our wound healing assay showed that over-expression of Skp2 caused increased numbers of cells migrating across the wound (Figure 4B). Consistently, our invasion assay showed that Skp2 promoted cell invasion in both glioma cells (Figure 4C). Strikingly, over-expression of Skp2 in combination with curcumin treatment led to lower numbers of migrated and invasive cells compared with curcumin treatment alone (Figure 4B and 4C). Next, we found that overexpression of Skp2 abrogated activation of p57 by curcumin (Figure 5A and 5B). These results provide evidence that curcumin exhibits anti-tumor activity through regulation of Skp2 and its target gene p57.

### Down-regulation of Skp2 by siRNA promotes curcumin-induced cell growth inhibition and apoptosis

**Figure 6:**
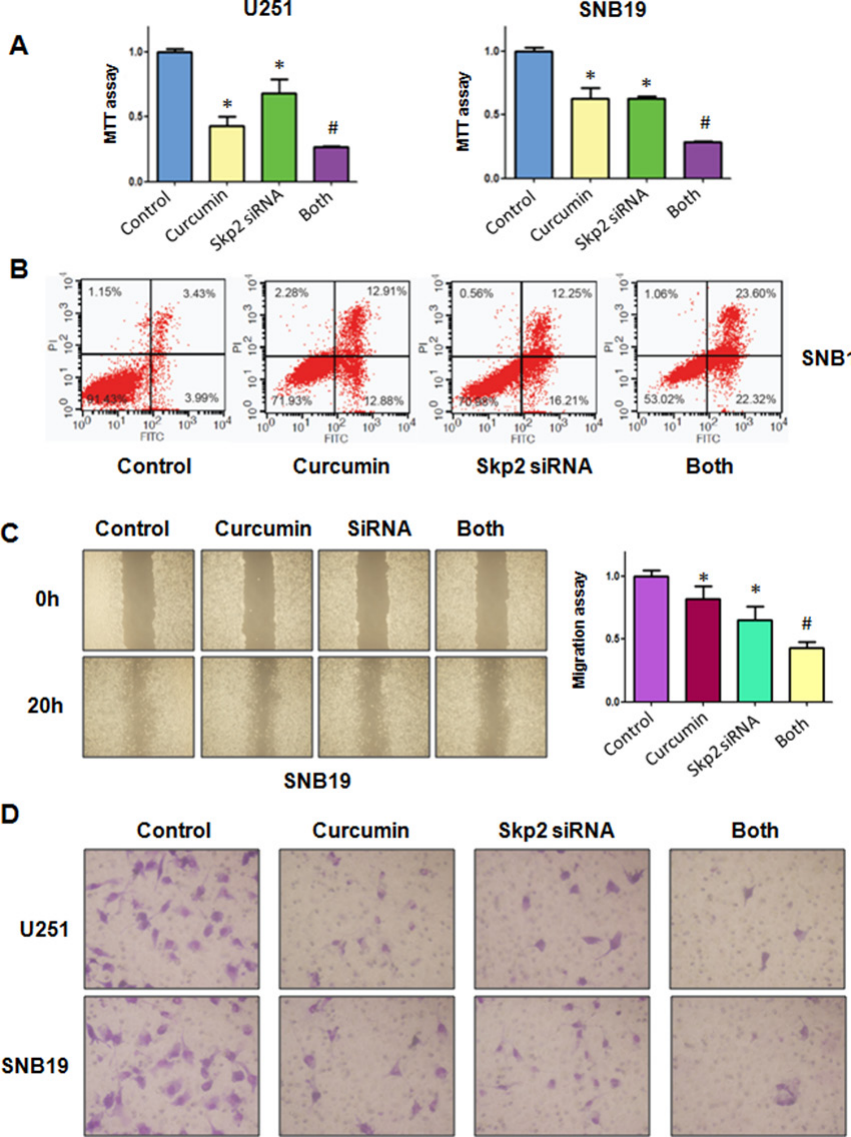
The effect of Skp2 downregulation on cell growth, apoptosis, migration, and invasion **A.** MTT assay was used to detect the effect of skp2 siRNA in combination with curcumin treatment on glioma cell proliferation. Both: curcumin + Skp2 siRNA. *P < 0.05, compared with control; ^#^*P* < 0.05 compared with curcumin treatment or Skp2 siRNA transfection. **B.** Apoptosis was detected by Flow cytometry in glioma cells with Skp2 siRNA transfection and curcumin treatment. **C.** The wound healing assay was conducted to detect the cell migration in glioma cells after Skp2 siRNA transfection and curcumin treatment. SiRNA: Skp2 siRNA; Both: curcumin + Skp2 siRNA. *P < 0.05, vs control; ^#^*P* < 0.05 vs curcumin treatment or Skp2 siRNA transfection. **D.** Invasion assay was performed in glioma cells after Skp2 siRNA transfection and curcumin treatment.

**Figure 7:**
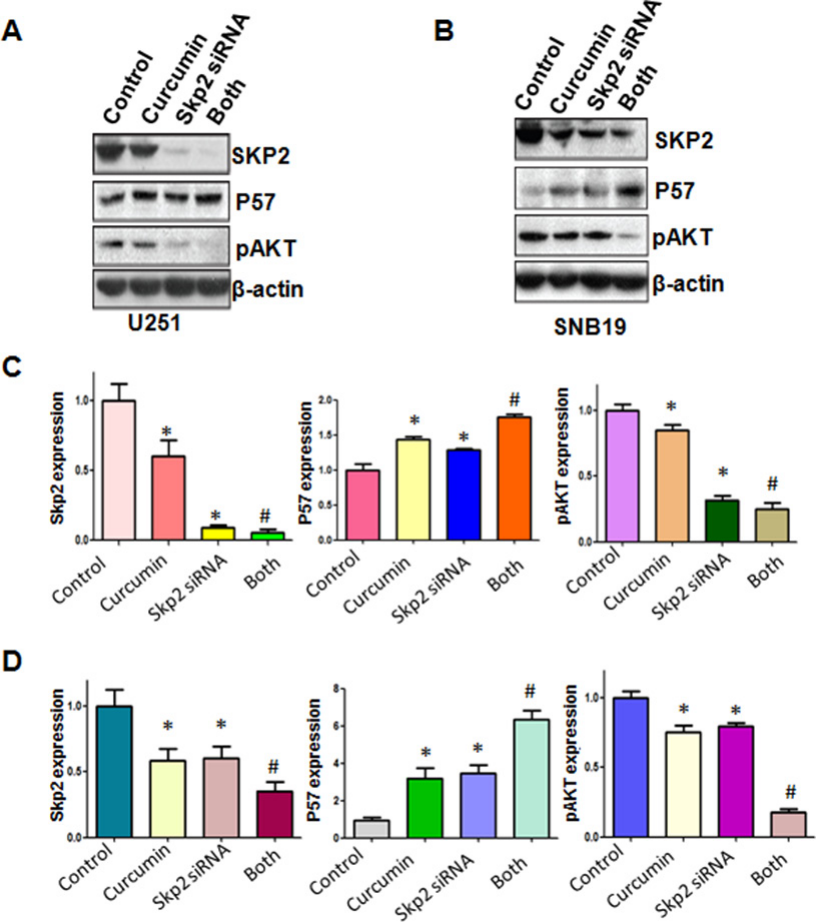
The expression of Skp2 and its targets was measured in Skp2 siRNA transfected glioma cells treated with curcumin **A–B.** The expression of Skp2 and its targets including p57 and pAkt was detected by western blotting in glioma cells with Skp2 siRNA transfection and curcumin treatment. **C–D.** Quantitative results are illustrated for panel A and panel B, respectively. *P < 0.05, vs control; ^#^*P* < 0.05 vs curcumin treatment or Skp2 siRNA transfection.

In line with the oncogenic role of Skp2, we found that the down-regulation of Skp2 expression significantly inhibited cell growth induced by curcumin (Figure 6A). Skp2 siRNA transfected cells were significantly more sensitive to spontaneous and curcumin-induced apoptosis (Figure 6B). Notably, we identified that depletion of Skp2 suppressed migration and invasion in glioma cells (Figure 6C and 6D). More importantly, depletion of Skp2 in combination with curcumin treatment retarded cell migration and invasion to a greater degree compared with curcumin alone or siRNA treatment alone (Figure 6C and 6D). Our results also showed that Skp2 siRNA inhibited pAkt activity and increased p57 expression; however, curcumin plus Skp2 siRNA inhibited pAkt activity and increased p57 expression to more degree compared to curcumin alone or siRNA transfection alone (Figure 7A–7D).

## DISCUSSION

In our present study, we found that curcumin inhibited cell growth and induced cell cycle arrest and apoptosis in U251 and SNB19 glioma cells. We further demonstrated that curcumin suppressed cell migration and invasion in glioma cell. Mechanistically, our findings demonstrated that curcumin exerts its anti-tumor functions through down-regulating Skp2 expression in glioma cells. These results revealed that curcumin could be an effective agent for treating glioma.

Multiple studies have demonstrated that Skp2 has an essential oncogenic function in the pathogenesis of glioma [33–35]. For example, Skp2 was diffusely or focally expressed in most glioblastomas, whereas Skp2 expression was absent or very low in well differentiated astrocytomas [33]. In keep of this concept, increased levels of Skp2 was observed in 31% GBM patients and was associated with shorter overall survival [35]. Therefore, inactivation of Skp2 could block development and progression of glioma. Indeed, down-regulation of Skp2 caused cell growth arrest and apoptosis in T98G glioblastoma cells [34]. In line with these findings, our data validated that inhibition of Skp2 retarded the cell growth, migration, and invasion, but induced cell apoptosis. Consistently, overexpression of Skp2 enhanced cell growth and cell motility in U251 and SNB19 cells.

Due to oncogenic function of Skp2 in glioma, inactivation of Skp2 could be a promising strategy for the treatment of glioma. To this end, several compounds have been discovered to inhibit the expression of Skp2. It has been reported that butylidenephthalide, isolated from Angelica sinensis, down-regulated Skp2 expression and subsequently increased p16 and p21 expressions, leading to cell senescence in human glioblastoma multiform [36]. Additionally, oncostatin M has been found to inhibit the Skp2 levels in glioblastoma cells [37]. Notably, Compound A has been discovered to block Skp2 E3 ligase activity [38]. It has been reported that Skp2 could be inhibited by caffeic acid phenethyl ester and curcumin in cancer cell lines [39–42]. Recently, Compound 25, also known as SZL-P1–41, was developed as a Skp2 inhibitor and inhibited Akt-mediated glycolysis and induced cellular senescence [31]. These inhibitors could have side effects in clinical trials. Due to non-toxic nature, inhibiting Skp2 by nature agents could be a safer approach for treating glioma. In this study, our results demonstrated that curcumin inhibited Skp2 expression in glioma cell lines. We further validated that curcumin exerts its anti-tumor function partly through down-regulation of Skp2. These findings indicated that curcumin may be a useful agent for achieving better treatment of human glioma.

Emerging evidence has shown that Akt can directly bind to Skp2 and phosphorylate its serine 72, leading to the translocation of Skp2 from the nucleus to cytoplasm and activation of oncogenic function of Skp2 [27, 43]. However, one study found that Akt drives Skp2 and FOXO1 to the cytoplasm [44, 45]. Further study demonstrated that Skp2 triggered Akt activation in human breast cancer [30]. In support of this note, we observed that Skp2 increased Akt activation in U251 and SNB19 glioma cells. Moreover, curcumin inhibited Skp2 target gene expression including Akt and p57. One study has shown that FoxM1 (Forkhead box protein M1) activated the expression of Skp2 in glioblastoma [46]. Curcumin was reported to suppress FoxM1 expression in human acute myeloid leukemia (AML) [47]. It is possible that curcumin down-regulated Skp2 expression due to inhibition of FoxM1. However, further investigation is required to explore this possibility. It is noteworthy to note that the therapeutic use of curcumin is hampered because of its rapid metabolism and poor absorption. It has been suggested that 2–20 μM curcumin for cancer cell lines, 50–200 mg/kg curcumin for mouse treatment, and 8–12 g/day for human trial were utilized [48–50]. Without a doubt, it is necessary to improve the bioavailable of curcumin and to overcome the blood-brain barrier. It is also required to determine whether curcumin exerts its anti-cancer function through inhibiting Skp2 expression in glioam mouse models *in vivo*. In conclusion, our studies provided that the inhibition of cell growth, induction of cell apotposis and cell cycle arrest, suppression of cell invasion and migration in glioma cell by curcumin could be partly through down-regulation of Skp2, suggesting that inhibiting Skp2 by curcumin could be an potential effective approach to treat glioma.

## MATERIALS AND METHODS

### Cell culture and reagents

Human glioma lines U251 and SNB19 were cultured in DMEM supplemented with 10% fetal bovine serum and 1% penicillin and streptomycin in a 5% CO2 atmosphere at 37°C. Primary antibodies for Skp2 and P57 were purchased from Santa Cruz Biotechnology (Santa Cruz, CA). Anti-pAkt (S473) was purchased from Cell Signaling Technology. All secondary antibodies were purchased from Thermo Scientific. Lipofectamine 2000 was purchased from Invitrogen. Monoclonal anti-β-actin, curcumin (CAS number 458-37-7, 99.5% curcumin) and MTT (3-(4, 5-dimethyl-2-thiazolyl)-2, 5-diphenyl-2-H-tetrazolium bromide) were obtained from Sigma-Aldrich (St. Louis, MO). Curcumin was dissolved in DMSO to make a 30 mM stock solution and was added directly to the media at different concentrations. Cells were treated with 0.1% DMSO as the control group.

### MTT assay

Cells were seeded at 5 × 10^3^ cells/well in 96-well plate for 24 h and treated with different concentrations of curcumin. After 48 h and 72 h, 10μl of the MTT (5 mg/ml) solution was added to each well and incubated for 4 h at 37°C. Then, the supernatant was absorbed and 100 μl DMSO was added to dissolve the MTT-formazan crystals. The absorption was measured by the microplate at 490 nm.

### Cell apoptosis analysis

Cells (1 × 10^5^ cells/well) were cultured in six-well plate overnight and treated with various concentration of curcumin for 48 h. Then, cells were harvested and washed with PBS, resuspended in 500μl binding buffer with 5 μl Propidium iodide (PI) and 5 μl FITC-conjugated anti-Annexin V antibody. Apoptosis was analyzed with a FACScalibur flow cytometer (BD, USA).

### Cell cycle analysis

Exponentially growing cells (2 × 10^5^ cells/well) were seeded in a 6-well plate overnight and then treated with 10 μM and 15 μM curcumin for 48 h. After 48 h, cells were collected and washed with cold PBS. Then, suspended cells with 70% cold alcohol were kept at 4°C overnight. Prior to analysis, the cells were washed with cold PBS, and re-suspended at 1 × 10^6^ cells/ml in PBS. Cells were incubated with 0.1mg/ml RNase I and 50 mg/ml Propidium iodide (PI) at 37°C for 30 min. Cell cycle was analyzed with a FACScalibur flow cytometer (BD, USA).

### Cell scratch assays

U251 cells and SNB19 cells were cultured in 6-well plate. After cells converged almost 100%, absorbed the supernatant and scratched the cells with a yellow pipette tips. Then washed the cells with PBS and added medium with curcumin. The scratched area was photographed with a microscope at 0 h and 20 h, respectively, as described before [51].

### Cell invasion assay

Cell invasion assay was conducted to test the invasive activity of U251 and SNB19 cells treated with curcumin or Skp2 transfection or combination [51]. Briefly, transfected cells were seeded in the upper chamber with 200 μl serum-free medium and there is 500 μl complete medium in the under chamber with the same concentration of curcumin. After incubation for 24 h, the membrane of the chamber was strained with Giemsa and photographed with a microscope.

### Transfection

Cells were seeded into 6-well plate and transfected with Skp2 cDNA or Skp2 siRNA or empty vector using lipofectamine 2000 following the instruction's protocol [52]. Skp2 siRNA: sense 5′-GGA GUG ACA AAG ACU UUG UTT-3′; antisense 5′-ACA AAG UCU UUG UCA CUC CTT-3′. After the transfection, the cells were subjected to further analysis as described under the results sections.

### Quantitative real-time reverse transcription-PCR analysis

The total RNA was extracted with Trizol (Invitrogen, Carlsbad, CA) and reversed-transcribed into cDNA by RevertAid First Strand cDNA Synthesis Kit. PCR were performed using Power SYBR Green PCR Master Mix and the results were calculated by 2-ΔΔCt method as described previously [52]. The primers used in the PCR reaction are: Skp2, forward primer (5′-GCT GCT AAA GGT CTC TGG TGT-3′) and reverse primer (5′-AGG CTT AGA TTC TGC AAC TTG-3′); GAPDH, forward primer (5′-ACC CAG AAG ACT GTG GAT GG-3′) and reverse primer (5′-CAG TGA GCT TCC CGT TCA G-3′).

### Western blotting analysis

The harvested cells were washed by PBS and lysed with protein lysis buffer. The concentrations of the proteins were tested by BCA Protein Assay kit (Thermo Scientific, MA). Same amount of protein samples were separated by electrophoresis in Sodium Dodecyl Sulfonate (SDS)-polyacrylamide gel and then transferred onto a Polyvinylidene Fluoride (PVDF) membrane, and then incubated with primary antibody at 4°C overnight. After washed with TBST for three times and incubated with second antibody at room temperature for one hour. Then the expression of protein was detected by electrochemiluminescence (ECL) assay.

### Statistical analysis

All statistical analyses were conducted using GraphPad Prism 4.0 (Graph Pad Software, La Jolla, CA). Student's *t-test* was performed to evaluate statistical significance. Results were presented as means ± SD. *P* < 0.05 was considered as statistically significant.
